# Spatial distribution, clustering patterns, and economic burden of comorbidities among hospitalised pulmonary tuberculosis patients in Guangdong province: a spatial epidemiological study based on inpatient big data

**DOI:** 10.7189/jogh.16.04284

**Published:** 2026-08-28

**Authors:** Lianrong Ji, Liang Chen, Yunlian Xue, Yue Wu, Huizhong Wu, Xue Jiang, Guihao Liu, Xiaowan Wang, Shasha Yuan, Lin Zhou

**Affiliations:** 1 School of Health Management, Southern Medical University, Guangzhou, China; 2 Department of Medical Insurance Affairs, Guangdong Provincial People's Hospital, Guangdong Academy of Medical Sciences, School of Health Management, Southern Medical University, Guangzhou, China; 3 State Key Laboratory of Respiratory Disease, Guangzhou Medical University Institute of Tuberculosis, Office of the Dean, Guangzhou Chest Hospital Affiliated to Guangdong Pharmaceutical University, Guangzhou, China; 4 Guangdong Provincial Center for Public Health Medicine, Guangzhou, China; 5 Department of Quality Control, Guangdong Provincial Center for Public Health Medicine, Guangzhou, China; 6 Office of the Party Committee, Guangdong Provincial People’s Hospital, Guangdong Academy of Medical Sciences, Southern Medical University, Guangzhou, China; 7 Department of Scientific Research, Guangdong Provincial People’s Hospital, Guangdong Academy of Medical Sciences, Southern Medical University, Guangzhou, China; 8 Division of Health Policy Research, Chinese Academy of Medical Sciences & Peking Union Medical College, Beijing, China; 9 Institute of Medical Information & Library, Chinese Academy of Medical Sciences & Peking Union Medical College, Beijing, China; 10 Office of the Dean, Guangdong Provincial People’s Hospital, Guangdong Academy of Medical Sciences, Southern Medical University, Guangzhou, China

**Keywords:** pulmonary tuberculosis, comorbidity, disease burden, spatial autocorrelation, Guangdong Province

## Abstract

**Background:**

Comorbidities may complicate the treatment of pulmonary tuberculosis and exacerbate both the clinical and economic burden. We aimed to examine comorbidity patterns, spatial clustering, and economic burden in hospitalised pulmonary tuberculosis (PTB) patients in Guangdong Province, supporting targeted prevention strategies.

**Methods:**

Based on the Hospital Discharge Records Database of Guangdong Province from 2016 to 2024, we collected data on hospitalised PTB patients to analyse the characteristics and temporal trends of comorbidity spectra. We used Global Moran’s I and local indicators of spatial association analyses to examine the spatial clustering patterns of major comorbidity pairs. Generalised linear models evaluated the impact of comorbidity count and spatial clustering patterns on economic burden among hospitalised PTB patients.

**Results:**

A total of 646,114 hospitalised PTB patients were included from 2016 to 2024, of whom 66.42% had at least one comorbidity. Hypertension (HTN), diabetes mellitus (DM), and chronic obstructive pulmonary disease (COPD) were the top three comorbidities among hospitalised PTB patients. Spatial analysis indicated that high-high clusters of PTB-HTN were mainly concentrated in the central Pearl River Delta; high-high clusters of PTB-DM in eastern Guangdong and low-low clusters in western Guangdong. High-high clusters of PTB-COPD were primarily located in eastern and northern Guangdong, while low-low clusters remained relatively stable in several cities of the Pearl River Delta region. Among hospitalised PTB patients, the number of comorbidities was significantly positively associated with both direct and indirect economic burden (*P* < 0.001), with economic burden progressively increasing as the number of comorbidities increased, and the impact of spatial clustering patterns on economic burden exhibited heterogeneity across different comorbidity types.

**Conclusions:**

The burden of comorbidities among hospitalised PTB patients in Guangdong Province is substantial, with significant spatial clustering characteristics closely linked to direct and indirect economic burden. It is recommended to incorporate regional comorbidity epidemiological characteristics into PTB comorbidity management and resource allocation optimisation. In addition, both direct medical costs and productivity losses should be considered in prevention and control strategies to reduce the socioeconomic burden associated with PTB.

Tuberculosis (TB) remains one of the world's deadliest infectious diseases, with an estimated 10.7 million (95% uncertainty interval (UI) = 9.9–11.5) people falling ill with TB globally in 2024, and 1.23 million (95% UI = 1.13–1.33) died from the disease [1]. The global estimated TB incidence rate per 100,000 population was 131 (95% UI = 122–141), with China accounting for 6.5% of global TB cases and ranking fourth worldwide. The large patient population in China signifies ongoing substantial challenges for TB prevention and control. Evidence from household cost surveys in 35 countries showed that the proportion of households experiencing catastrophic total costs (*i.e.* direct and indirect costs >20% of annual household expenditure or income) due to TB ranged from 13% to 92%, with a weighted average of 49%, and increased to 82% among those affected by drug-resistant TB (DR-TB) [2]. These costs prevent many patients from completing the full course of treatment from diagnosis to cure, adversely affecting their health and well-being while increasing the risk of disease transmission. The ‘compounded economic burden’ arising from TB comorbidities represents a critical issue requiring urgent quantification [3], as they not only increase the risk of developing TB but may also lead to poor treatment outcomes, reduced quality of life, and increased economic burden. The World Health Organization emphasises the need for routine assessment and management of TB comorbidities [4].

According to the surveillance data on notifiable infectious diseases in Guangdong Province, the number of reported TB cases remains persistently high, with pulmonary TB (PTB) consistently ranking among the top three notifiable infectious diseases in terms of incidence and mortality. As one of China's most economically developed regions, Guangdong Province faces significant challenges in TB prevention and control due to its dense population and large proportion of migrants. A study conducted in Shenzhen showed that the risk of cross-district TB transmission among the migrant population was 3.41 times higher than among residents [5]. A large migrant population constitutes a high-risk group for TB, and challenges such as cross-regional healthcare-seeking, difficulties in managing treatment adherence, and complex disease presentations greatly increase the difficulty of controlling disease transmission and ensuring standardised patient management [6,7]. *Mycobacterium Tuberculosis* causes TB, a chronic infectious disease which typically affects the lungs (PTB) and is both preventable and curable [8]. Previous research has confirmed that PTB exhibits significant spatial and temporal heterogeneity [9,10], suggesting that TB control must transcend a purely biomedical perspective and delve into analysis from the viewpoints of social determinants and geographic space.

However, existing research on PTB comorbidities has notable limitations. First, the studies are mostly based on single-centre data, lacking systematic analysis of spatial distribution characteristics at the provincial level. Second, small-scale surveys struggle to reflect macro-level clustering patterns and are limited in revealing underlying social and environmental influencing factors [7]. Third, the economic dimension is often missing – the ‘compounded burden’ that comorbidities impose on the health system has not been adequately quantified. Notably, the spatial clustering of diseases often reflects shared social, economic, or environmental driving mechanisms. Therefore, identifying comorbidity cluster areas is significant for developing regionalised and precision public health intervention strategies.

To address these gaps, we aimed to systematically analyse the comorbidity spectrum of hospitalised PTB patients in Guangdong Province and use spatial analysis methods to identify their spatial distribution patterns and clustering characteristics. We also sought to quantitatively assess the impact of different comorbidity statuses on the inpatient economic burden among hospitalised PTB patients and analyse its association with spatial clustering patterns. Our results could provide a scientific basis for formulating regionalised, precision, and economic risk-sensitive comprehensive TB prevention and control strategies

## METHODS

### Data sources

We included inpatient medical record front-page data for all hospitalised patients diagnosed with PTB in Guangdong Province from 1 January 2016 to 31 December 2024. The ICD-10 codes A15, A16, and A19 were used to identify PTB cases from any diagnostic field in the inpatient record. For patients coded as A19, we restricted the inclusion to cases whose diagnosis name contained the term ‘pulmonary’ in the diagnosis name field. We sourced the data from the Guangdong Provincial Health Statistics Information Network Direct Reporting System, covering all public and private medical institutions in the province (*e.g.* general hospitals, maternal and child health hospitals, and specialised disease prevention and treatment hospitals). Data from 2016–2017 covered inpatient records from all medical institutions in the province except primary care facilities (*e.g.* community health service centres and township health centres). From 2018 onwards, data were expanded to include inpatient records from all medical institutions province-wide, including primary care facilities, achieving full coverage of all inpatients in the province. We collected diagnosis codes, total hospitalisation expenses, out-of-pocket expenses, present address, unique patient identifiers, age, sex, admission route, surgical procedure level, case type, actual hospitalisation days, hospital level, and discharge date. For multiple admissions of the same patient within the same year, we applied the ‘latest diagnosis priority’ principle, retaining only the most recent hospitalisation diagnostic information based on the unique identifier to avoid duplicate counting interference. Observations with incomplete data were excluded to ensure data quality, resulting in a final cohort of 646,114 hospitalised PTB patients. According to the ‘institution type’ field in the inpatient medical record database, there were no hospitalised PTB patient records from primary healthcare facilities (B- and C-type facilities). We analysed all patient-related data within the internal server environment hosting the data; raw data were managed in a closed loop throughout the process, posing no risk of leakage.

### Variable definition and processing

Following a systematic literature review, we included 20 diseases as ‘PTB comorbidities’: hypertension (HTN), disorders of lipoprotein metabolism and other lipidaemias, diabetes mellitus (DM), malignant tumour, rheumatoid arthritis, coronary atherosclerotic heart disease, viral hepatitis, liver cirrhosis, chronic obstructive pulmonary disease (COPD), cerebrovascular diseases, atherosclerosis, mental and behavioural disorders, hyperuricaemia, bronchiectasis, hypoproteinaemia, asthma, malnutrition, emphysema, human immunodeficiency virus disease, and chronic kidney disease. All comorbidities were identified based on ICD-10 codes (Table S1 in the **Online Supplementary Document**), which were identified from all available diagnosis fields recorded in the inpatient medical record. For the analysis, we categorised the participants as having ‘no comorbidity’, ‘only one comorbidity’, ‘only two comorbidities’, and ‘≥3 comorbidities’.

Based on the administrative division codes and the ‘Guangdong Map (Administrative Division Version)’ published *via* the Standard Map Service Subsystem of the Department of Natural Resources of Guangdong Province, the variable ‘region’ was defined by dividing Guangdong Province into four areas: the Pearl River Delta, Eastern Guangdong, Western Guangdong, and Northern Guangdong. We also collected the participants' surgery status, dividing the participants into those who have undergone surgery and those who have not. Actual hospitalisation days were considered as work-loss days. The value of labour was represented by the average daily gross domestic product (GDP) per capita, used as a proxy indicator for the loss per workday. Productivity weights, based on current international practice, were assigned to four age groups of hospitalised patients (0–14, 15–44, 45–59, and ≥60 years) as 0, 0.75, 0.8, and 0.1, respectively, to adjust for productivity differences due to labour force variations across age groups. Total hospitalisation expenses quantified direct economic burden. Due to data availability constraints, the indirect economic burden was estimated using the human capital approach and reflects productivity losses associated with hospitalisation days. We calculated it as: indirect economic burden = actual hospitalisation days × daily GDP per capita × productivity weight

Daily GDP per capita was derived from per capita GDP data for each city published in the Guangdong Statistical Yearbook. Due to the long study period, total hospitalisation expenses, out-of-pocket expenses, and daily GDP per capita were inflation-standardised using the 2024 Consumer Price Index as the baseline (100.0%), with the following annual adjustment factors: 2016: 102.3%, 2017: 101.5%, 2018: 102.2%, 2019: 103.4%, 2020: 102.6%, 2021: 100.8%, 2022: 102.2%, 2023: 100.4%.

### Statistical analysis

We presented categorical data as numbers (%) and performed comparisons between groups using the χ^2^ test or Fisher exact test. For ordinal categorical variables and their relationship with binary outcomes, the χ^2^ test for trend was further employed based on the initial χ^2^ test. Non-normally distributed continuous data were presented as median (interquartile range), and comparisons between groups were performed using the Mann−Whitney U test or Kruskal−Wallis rank-sum test.

We used generalised linear models (GLMs) to examine the associations between comorbidity burden and economic burden. All models were constructed using a Gamma distribution with a log-link function to account for the right-skewed distribution of economic burden data. We reported the results as exponentiated regression coefficients (exp(*β*)) with corresponding 95% confidence intervals.

To progressively evaluate the independent effects after adjustment for potential confounding factors, we constructed GLMs using a stepwise adjustment strategy. Model 1 was the baseline model and included year, disease clustering variables (PTB-HTN cluster, PTB-DM cluster, PTB-COPD cluster), and number of comorbidities to assess the crude effect of disease clustering patterns on costs while controlling for temporal trends. Model 2 additionally included demographic variables (age and sex). Model 3 was fully adjusted, further adding clinical characteristic variables (*i.e.* case type, surgery status, and actual hospitalisation days), admission route, hospital level, and region.

Global and local Moran’s I statistics were used to evaluate the spatial distribution characteristics of major comorbidity combinations among hospitalised PTB patients across prefecture-level cities in Guangdong Province and to identify hotspot areas (high-high clusters), cold spots (low-low clusters), and spatial outliers. Moran’s I values ranged from −1 to 1, with positive values indicating positive spatial autocorrelation and values closer to 1 indicating stronger spatial clustering. We also calculated Z statistics to determine whether the prevalence distributions of comorbidity combinations were spatially random, with the statistical significance set at |Z| > 1.96 and *P* < 0.05.

We used local indicators of spatial association (LISA) cluster maps to visualise spatial clustering patterns. We adopted a Queen contiguity spatial weight matrix. Two prefecture-level cities were defined as neighbours if they shared either a common boundary or a vertex (wij = 1); otherwise, wij = 0. The spatial weight matrix was row-standardised. Among the 21 prefecture-level cities in Guangdong Province, the minimum number of neighbours was 1, the maximum was 6, and the mean number of neighbours was 3.71. For boundary cities with relatively few neighbours, conditional random permutation tests were applied to preserve the true adjacency structure, thereby reducing the influence of boundary effects on the LISA results.

We evaluated the statistical significance of local Moran’s I using conditional permutation tests with 999 random permutations to generate inferential statistics. Because repeated annual LISA analyses across multiple disease combinations and cities may increase the probability of false-positive local clusters, no additional formal multiple-comparison correction methods, such as Bonferroni correction or false discovery rate adjustment, were applied. Therefore, we interpreted the spatial analyses as exploratory analyses, and short-term annual clustering anomalies were interpreted cautiously.

### Sensitivity analysis

We conducted sensitivity analyses to evaluate the robustness of the study findings. To assess the stability of the spatial autocorrelation results, we repeated global Moran’s I and LISA analyses using a Rook contiguity matrix (shared boundaries only) instead of the Queen contiguity matrix employed in the primary analysis. For the economic burden analyses, we performed several additional analyses. First, GLMs were fitted using the original cost data to verify the robustness of the estimates. Second, we repeated the analyses using all hospitalisation records of patients with PTB to assess the potential influence of repeated hospitalisations on the results. Given that length of stay (LOS) may partially mediate the relationship between comorbidity burden and hospitalisation costs, we estimated an additional GLM for total hospitalisation costs without adjustment for LOS to evaluate the potential impact of overadjustment. Finally, to account for potential within-cluster correlation among patients nested within the same city-year units, we applied city-year clustered robust standard errors in sensitivity analyses while maintaining the original model specification.

We used SPSS, version 20.0 (IBM Corp., Armonk, New York, USA) for all statistical analyses. Spatial autocorrelation analysis and LISA cluster maps were performed using GeoDa, version 1.22 (GeoDa Center for Geospatial Analysis and Computation, University of Chicago, Chicago, Illinois, USA). Statistical significance was evaluated using 999 random permutations with a significance level of α = 0.05.

## RESULTS

### Characteristics of hospitalised PTB patients

From 2016 to 2024, a total of 646,114 patients hospitalised with PTB were included in the province, including 3755 patients with DR-TB. Overall, male patients constituted a larger proportion. The age distribution of hospitalised PTB patients was predominantly ≥60 years, while DR-TB patients were mainly aged <45 years (**Table 1**). From 2016 to 2024, the number of hospitalised PTB patients showed a significant decrease in 2020, followed by a relatively slow upward trend. There was an increasing trend of DR-TB patients (Figure S1, Panel A in the **Online Supplementary Document**).

**Table 1:** Basic characteristics of hospitalised patients, n (%)

	PTB	DR-TB
**Sex**		
Male	476,826 (73.80)	2745 (73.10)
Female	169,288 (26.20)	1010 (26.90)
**Age group in years**		
<45	183,637 (28.42)	1464 (38.99)
45–60	167,749 (25.96)	1138 (30.31)
≥60	294,728 (45.62)	1153 (30.71)

PTB – pulmonary tuberculosis, DR–TB – drug-resistant tuberculosis

Regarding expenditure among hospitalised PTB patients, the proportion of drug costs significantly declined, dropping continuously from 33.00% to 13.31%, a decrease of approximately 50%. Median out-of-pocket expenses remained relatively stable before 2020, increased to CNY 3301.91 in 2020, and then gradually declined overall to CNY 2786.48 by 2024 (Figure S1, Panel B in the **Online Supplementary Document**). The median out-of-pocket expenditure remained relatively stable before 2020. Similarly, the proportion of out-of-pocket expenditure remained relatively stable before 2020 and increased to 38.09% in 2020. The median total hospitalisation expenses fluctuated, rising from CNY 8042.82 to a peak of CNY 9455.36 in 2021, then falling back to CNY 8379.42 in 2024. The median indirect economic burden fluctuated, increasing from CNY 463.17 to CNY 589.16 in 2021, then decreasing to CNY 495.32 in 2024.

### Comorbidity profile of hospitalised PTB patients

#### Distribution of comorbidity types

From 2016 to 2024, a total of 429,176 (66.42%) hospitalised PTB patients had at least one comorbidity. Among them, 184,623 (28.57%) had only one comorbidity, 124,939 (19.34%) had two comorbidities, and 119,614 (18.51%) had ≥3 comorbidities. Among the 20 included comorbidities, HTN (n = 116,915; 18.10%) and DM (n = 109,133; 16.89%) were the most common, followed by COPD (11.40%), hypoproteinaemia (11.16%), and bronchiectasis (10.54%). Other major comorbidities included cerebrovascular diseases (9.84%), emphysema (9.40%), hyperuricaemia (8.71%), and coronary atherosclerotic heart disease (6.49%) (Table S2 in the **Online Supplementary Document**). Overall, the annual number of patients with at least one comorbidity increased from 42,973 in 2016 to 52,206 in 2024, despite a temporary decline in 2020; it peaked at 52,381 in 2023 before decreasing slightly in 2024.

#### Differences in comorbidity spectrum by age group

The comorbidity spectrum of hospitalised PTB patients differed significantly across age groups (Table S2 in the **Online Supplementary Document**). Within the <45 years group, endocrine and metabolic diseases were the most common, including hyperuricaemia (10.87%) and DM (5.98%), with mild respiratory diseases (*e.g.* bronchiectasis 6.00%). Within the 45–60 years group, DM (23.47%) was the most prevalent, with a marked increase in circulatory system diseases such as HTN (14.28%) and atherosclerosis (4.29%) and respiratory diseases like COPD (5.66%) and bronchiectasis (9.97%). The ≥60 years group exhibited a typical pattern of multiple coexisting conditions, with significantly higher prevalence of severe chronic diseases such as HTN (30.26%), COPD (21.51%), and DM (19.94%), representing the heaviest medical burden.

#### Sex and age differences in comorbidity rates

Comorbidity rate was significantly higher for males (70.06%) compared to females (56.18%) hospitalised PTB patients (χ^2^ = 10790.154, *P* < 0.05). The comorbidity rate increased with age (χ^2^ trend = 105598.17, *P* < 0.05) (Table S3 in the **Online Supplementary Document**).

### Spatiotemporal distribution characteristics of major PTB comorbidity combinations

#### Temporal distribution characteristics of comorbidity rates

From 2016 to 2024, the overall comorbidity rate among hospitalised PTB patients in Guangdong Province showed a continuous upward trend. The number of patients with comorbidities increased from 42,973 (58.55%) in 2016 to 52,206 (74.86%) in 2024 (Figure S2, Panel A in the **Online Supplementary Document**). The prevalence of comorbidities in the <45 years group increased from 30.33% in 2016 to 46.94% in 2024, while that in the 45–59 years group rose from 60.22% to 76.31%. Among patients aged ≥60 years, the comorbidity prevalence increased from 78.08% in 2016 to 88.39% in 2024.

The comorbidity rate of PTB-HTN increased steadily, with the number of cases rising from 10,393 (14.16%) in 2016 to 15,622 (22.40%) in 2024. The PTB-HTN comorbidity rate varied across cities. Regions with high comorbidity prevalence were primarily concentrated in eastern Guangdong and several cities within the Pearl River Delta. Jiangmen exhibited the highest comorbidity prevalence, reaching 32.5% in 2024. Meizhou (28.1%), Shantou (27.5%), Shanwei (27.4%), and Zhongshan (26.8%) also consistently demonstrated relatively high prevalence levels in 2024. In contrast, northern Guangdong cities such as Shaoguan (18.5%), Qingyuan (23.7%), and Heyuan (21.9%) showed moderate prevalence levels in 2024, and Shenzhen, Dongguan, Zhanjiang, Maoming, and Yangjiang consistently exhibited relatively low prevalence rates, all <17% in 2024. In Guangzhou, the prevalence increased steadily from 20.5% in 2016 to 24.1% in 2024, although it remained lower than that observed in peak-prevalence cities such as Jiangmen and Meizhou. Overall, the spatial distribution of PTB-HTN comorbidity prevalence was characterised by a pattern of ‘higher in the east and lower in the west’, with substantial intra-regional heterogeneity within the Pearl River Delta. (Figure S2, Panel B in the **Online Supplementary Document**).

The number of hospitalised PTB patients with DM comorbidity increased from 9388 (12.79%) in 2016 to 14,702 (21.08%) in 2024. The prevalence of PTB-DM comorbidity among hospitalised PTB patients showed an increasing trend across all cities. High-prevalence areas were relatively dispersed, with leading cities distributed across eastern Guangdong (*e.g.* Heyuan, Jieyang, Meizhou, and Chaozhou), the Pearl River Delta (*e.g.* Huizhou, Zhuhai, and Guangzhou), and western Guangdong (*e.g.* Yangjiang). In 2024, the prevalence reached 26.1% in both Heyuan and Jieyang, 25.3% in Meizhou, and 24.3% in Huizhou. Chaozhou reported a prevalence of 25.4% in 2023, while Jieyang showed approximately 24.4% during the same year. In contrast, relatively low prevalence rates were observed in Zhanjiang, Maoming, Dongguan, and Qingyuan, all of which remained <19% in 2024. Overall, the spatial distribution of PTB-DM comorbidity demonstrated a pattern of ‘multiple high-prevalence centres with localised clustering’ (Figure S2, Panel C in the **Online Supplementary Document**).

The prevalence of PTB-COPD comorbidity among hospitalised PTB patients showed slight overall fluctuations, ranging from 10.26% to 12.40%, with heterogeneous temporal trends observed across cities. In several cities, the prevalence initially declined and subsequently increased. For example, in Guangzhou, the prevalence decreased from 8.6% in 2016 to 5.7% in 2021, before rising to 6.9% in 2024, although it remained below the 2016 level. In Jiangmen, the prevalence fluctuated slightly from 10.3% in 2016 to 10.5% in 2020, followed by a continuous increase to 18.2% in 2024. Shaoguan demonstrated a fluctuating upward trend from 20.0% to 24.4% during the study period. High-prevalence cities were primarily concentrated in northern Guangdong (*e.g.* Shaoguan and Qingyuan) and eastern Guangdong (*e.g.* Meizhou and Shanwei). In 2024, the prevalence reached 24.4% in Shaoguan, 23.8% in Meizhou, and 20.7% in Qingyuan. In contrast, Pearl River Delta cities such as Shenzhen, Zhuhai, Dongguan, Zhongshan, and Guangzhou generally exhibited relatively low prevalence rates, all <7% in 2024, except Jiangmen (18.2%). Overall, the spatial distribution of PTB-COPD comorbidity prevalence was characterised by a pattern of higher prevalence in northern and eastern Guangdong and lower prevalence in the Pearl River Delta and western Guangdong (Figure S2, Panel D in the **Online Supplementary Document**).

#### Spatial statistical analysis of comorbidity rates for major comorbidity groups

Spatial autocorrelation analysis showed that the PTB-HTN comorbidity rate exhibited statistically significant positive spatial autocorrelation across all study years (all *P* < 0.05), indicating a non-random spatial clustering distribution. The global Moran’s I value reached its highest level in 2024 (I = 0.401, *P* = 0.002), suggesting a relatively strengthened spatial clustering pattern in the later study period (Table S4 in the **Online Supplementary Document**).

**Figure 1:**
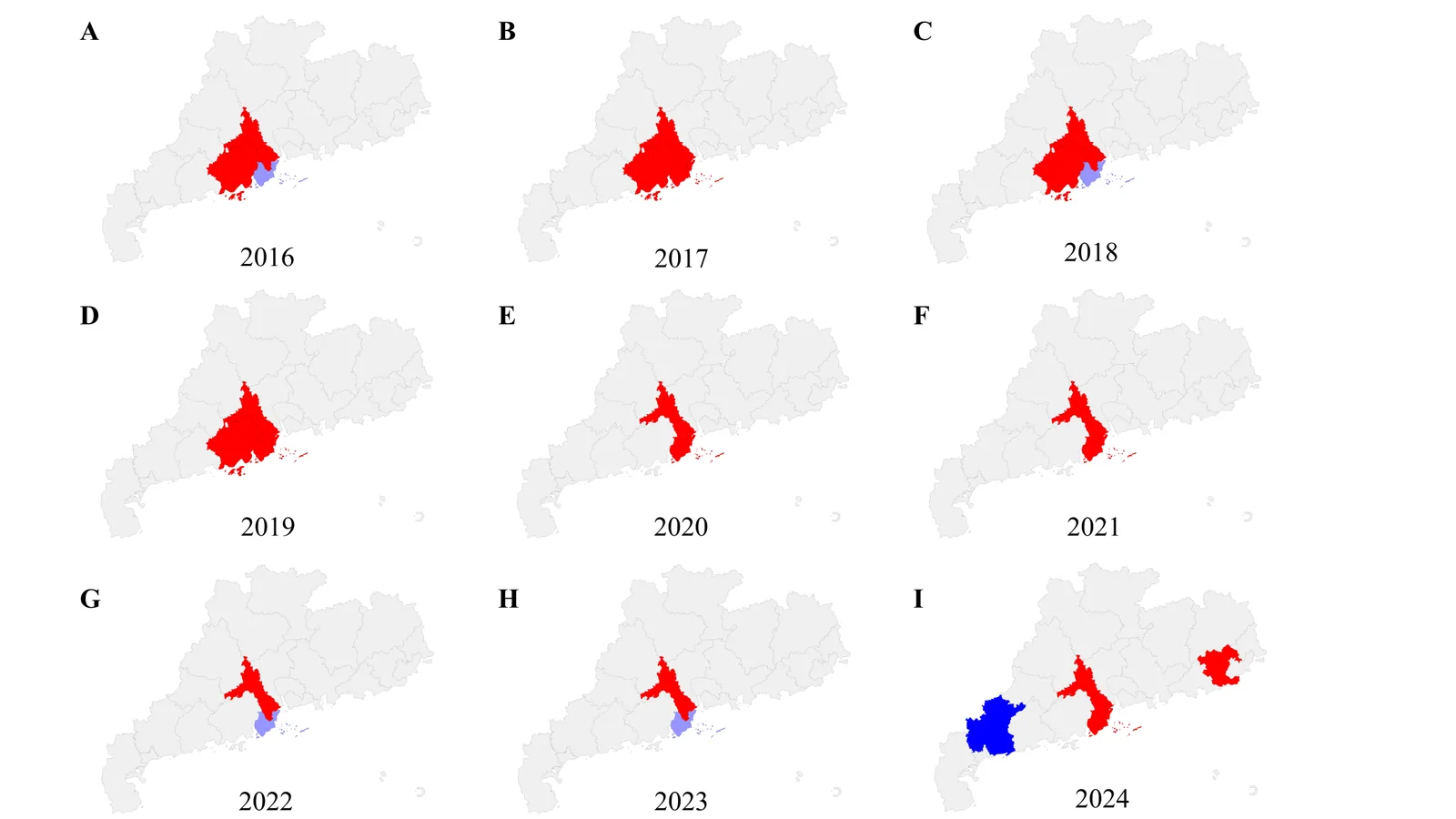
LISA cluster map of the comorbidity prevalence of HTN among hospitalised PTB patients, 2016–2024 (Panels A–I, respectively). HTN – hypertension, LISA – local indicators of spatial association, PTB – pulmonary tuberculosis.

The LISA cluster maps demonstrated that the local spatial association patterns of PTB-HTN comorbidity rates were mainly characterised by high-high (HH) and low-high (LH) clusters, whereas low-low (LL) clusters were observed only in 2024. From 2016 to 2019, statistically significant HH clusters were stably concentrated in the core cities of the Pearl River Delta, including Foshan, Jiangmen, and Zhongshan. Since 2020, Jiangmen no longer exhibited an HH clustering pattern. Zhuhai was identified as an HH cluster during 2019–2021 and again in 2024 but shifted to an LH cluster in 2022–2023. In 2024, Jieyang newly emerged as an HH cluster, together with Foshan, Zhongshan, and Zhuhai, forming a statistically significant hotspot region. The LH clustering was mainly observed in Zhuhai (in 2016, 2018, 2022, and 2023), whereas LL clustering was detected only in Maoming in 2024, indicating the formation of a localised cold spot area (**Figure 1**).

Spatial autocorrelation analysis showed that the PTB-DM comorbidity rate exhibited statistically significant positive spatial autocorrelation across all study years (all *P* < 0.05), with Moran’s I values remaining at a relatively high level overall. Moran’s I reached 0.494 in 2020 and 0.500 in 2022, indicating that the spatial clustering pattern was relatively stable and persistent throughout the study period (Table S4 in the **Online Supplementary Document**).

**Figure 2:**
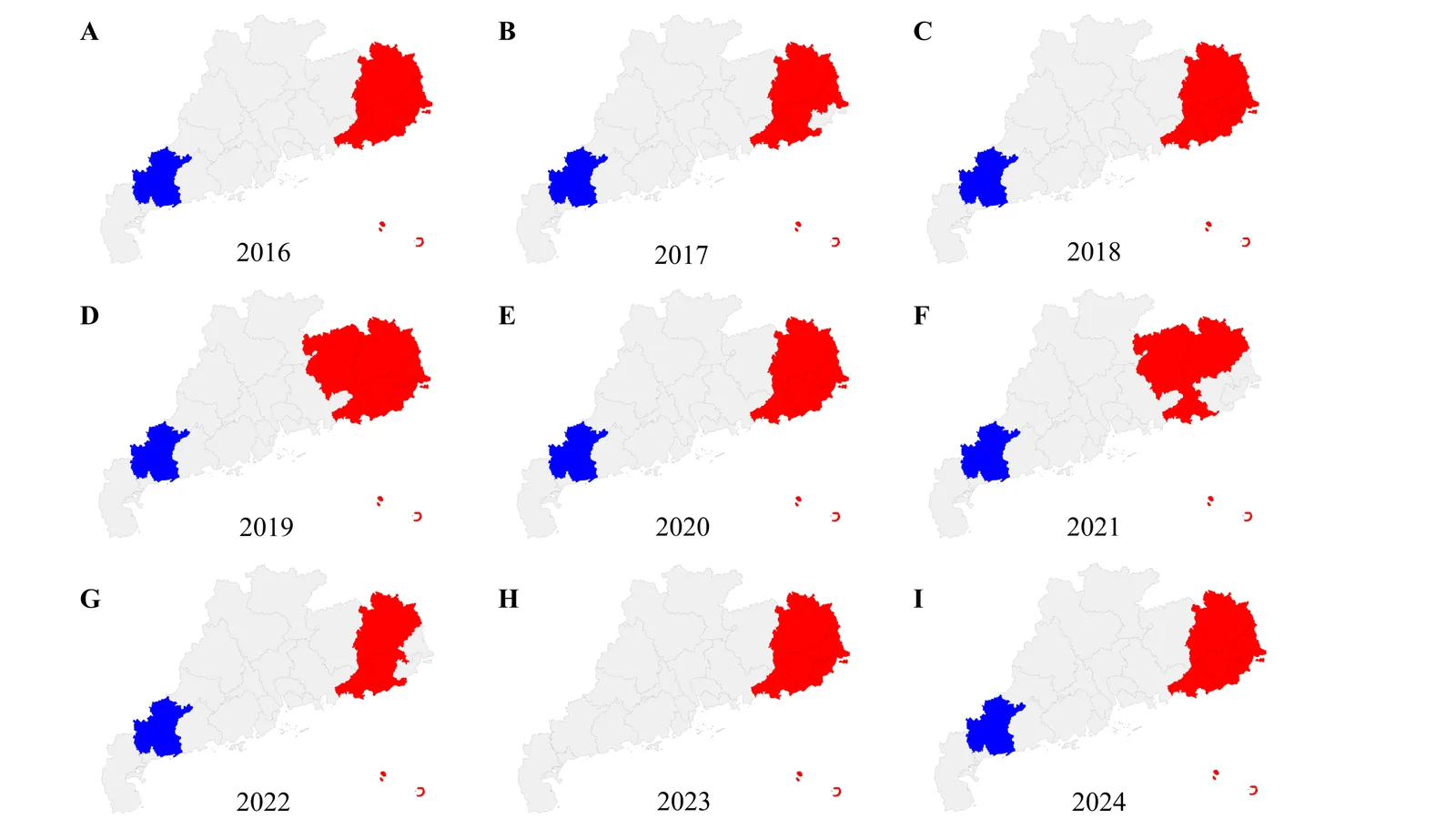
LISA cluster map of the comorbidity prevalence of DM among hospitalised PTB patients, 2016–2024 (Panels A–I, respectively). DM – diabetes mellitus, LISA – local indicators of spatial association, PTB – pulmonary tuberculosis.

The LISA cluster maps demonstrated that statistically significant HH clusters of PTB-DM comorbidity rates were mainly concentrated in eastern Guangdong, whereas LL clusters were persistently distributed in western Guangdong. From 2016 to 2020, Meizhou, Chaozhou, Jieyang, Shanwei, and Shantou formed a relatively stable HH clustering pattern; however, Shantou did not exhibit HH clustering in 2017, and Heyuan temporarily entered the HH cluster in 2019. In 2021, the HH clustering area contracted to Meizhou, Heyuan, and Shanwei. In 2022, the HH clusters mainly included Meizhou, Jieyang, and Shanwei. During 2023–2024, the HH clustering area expanded again to Meizhou, Chaozhou, Jieyang, Shanwei, and Shantou, showing a spatial pattern largely similar to that observed in the early study period. Statistically significant LL clustering was consistently observed in Maoming during 2016–2022 and again in 2024 (**Figure 2**).

Spatial autocorrelation analysis showed that the PTB-COPD comorbidity rate exhibited statistically significant positive spatial autocorrelation from 2016 to 2023 (all *P* < 0.05), whereas the global Moran’s I in 2024 did not reach statistical significance (I = 0.215, *P* = 0.061). Moran’s I reached its highest value in 2018 (I = 0.456, *P* = 0.002), suggesting that the degree of spatial clustering initially strengthened and subsequently weakened over time (Table S4 in the **Online Supplementary Document**).

**Figure 3:**
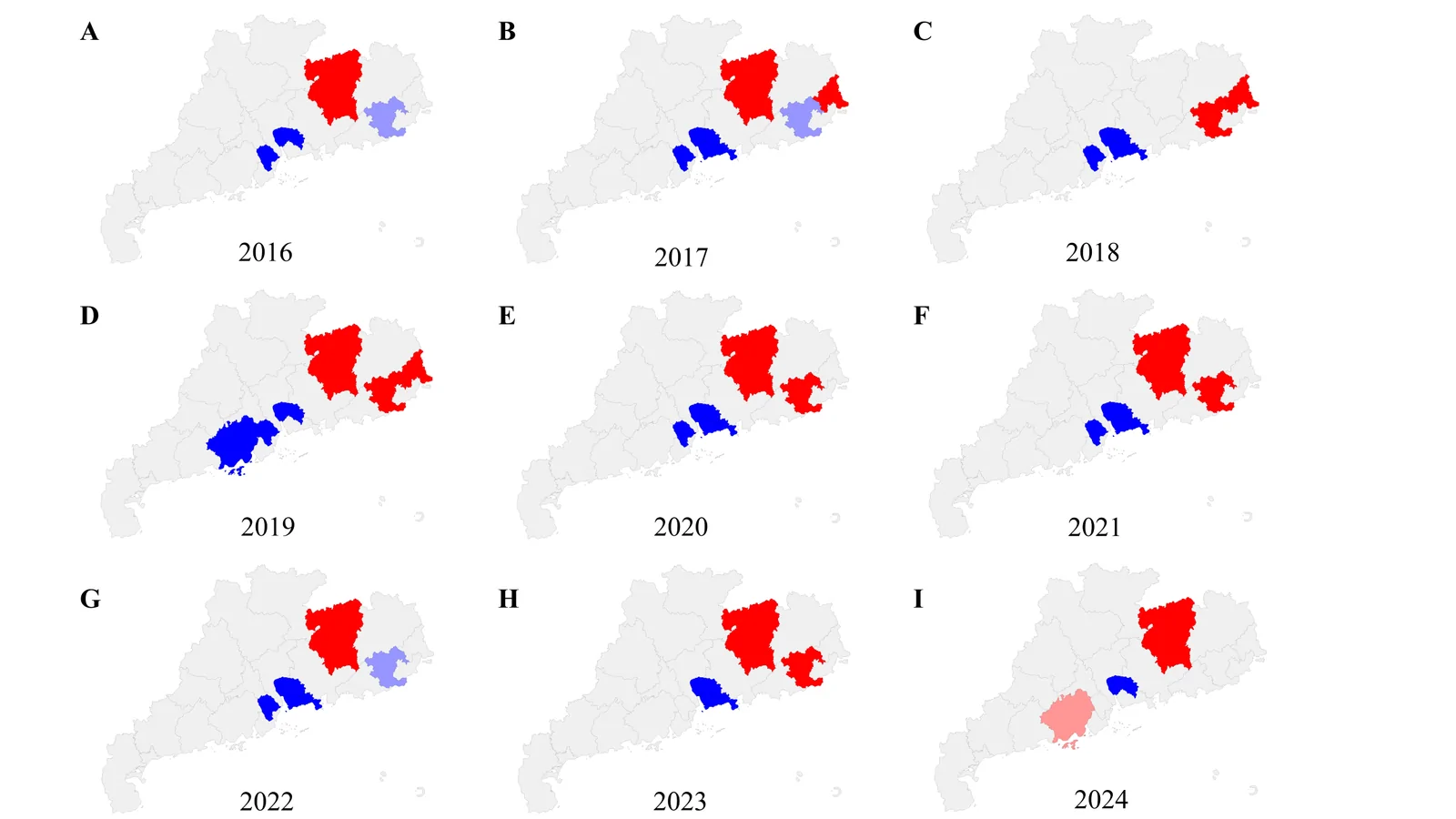
LISA cluster map of the comorbidity prevalence of COPD among hospitalised PTB patients, 2016–2024 (Panels A–I, respectively). COPD – chronic obstructive pulmonary disease, LISA – local indicators of spatial association, PTB – pulmonary tuberculosis.

The LISA cluster maps demonstrated that the PTB-COPD comorbidity rate exhibited multiple local spatial association patterns, including HH, LL, LH, and high-low (HL) clusters. The HH clusters were mainly distributed in parts of eastern and northern Guangdong. Among these, Heyuan was the most persistent HH cluster, exhibiting HH clustering in 2016, 2017, and 2020–2024. Chaozhou showed HH clustering during 2017–2019. Jieyang exhibited HH clustering during 2018–2021 and again in 2023 but shifted to an LH cluster in 2022 and no longer demonstrated statistically significant local clustering in 2024. In contrast, LL clusters were stably distributed in the Pearl River Delta region, mainly including Dongguan, Zhongshan, and Shenzhen. Specifically, LL clustering was observed in Dongguan and Zhongshan in 2016; in Dongguan, Zhongshan, and Shenzhen during 2017–2018 and 2020–2022; in Dongguan and Shenzhen in 2023; and only in Dongguan in 2024. LH clustering was mainly observed in Jieyang (in 2016–2017 and 2022). In 2024, Jiangmen exhibited HL clustering for the first time (**Figure 3**).

### Multivariable analysis

Univariate analysis showed that 12 factors – sex, age group, admission route, surgery status, case type, actual hospitalisation days, hospital level, region, number of comorbidities, PTB-HTN comorbidity rate cluster, PTB-DM comorbidity rate cluster, and PTB-COPD comorbidity rate cluster – had statistically significant associations with both direct and indirect economic burdens (*P* < 0.05).

All variables with statistical significance in the univariate analysis were included in the GLMs for multivariable analysis. Regarding variable settings in the GLM, ‘number of comorbidities’ used ‘no comorbidity’ as the reference group. For the spatial clustering patterns of the top three comorbidity diseases (PTB-HTN, PTB-DM, PTB-COPD), ‘no significant spatial clustering’ was used as the reference group. Since the dependent variable in the indirect economic burden model was calculated using ‘hospitalisation days’, this variable was not re-included as an independent variable. To evaluate the potential mediating effect of length of hospital stay, an additional model excluding hospitalisation days was further constructed in the sensitivity analysis of direct economic burden.

In the fully adjusted Model 3, comorbidity count was significantly associated with both direct and indirect economic burden (all *P* < 0.001), with a clear dose-response relationship observed. Compared with patients without comorbidities, those with one (exp(*β*) = 1.083), two (exp(*β*) = 1.145), and three or more (exp(*β*) = 1.220) comorbidities had progressively higher direct economic burden (*P* < 0.001). Similarly, compared to those without comorbidities, patients with one (exp(*β*) = 1.146), two (exp(*β*) = 1.217), and three or more (exp(*β*) = 1.302) comorbidities had progressively higher indirect economic burden (*P* < 0.001). In sensitivity analyses excluding LOS, direct economic burden remained significantly higher among patients with one (exp(*β*) = 1.192), two (exp(*β*) = 1.308), and three or more (exp(*β*) = 1.449) comorbidities than among those without comorbidities (*P* < 0.001). The larger effect estimates observed in the sensitivity model suggest that LOS may partially mediate the association between comorbidity burden and direct economic burden (**Table 2**, **Table 3**; Table S5 in the **Online Supplementary Document**).

**Table 2:** GLM analysis of direct economic burden for hospitalised PTB patients

	Model 1		Model 2		Model 3	
	**exp(*β*) (95% CI)**	***P*-value**	**exp(*β*) (95% CI)**	***P*-value**	**exp(*β*) (95% CI)**	***P*-value**
**Number of comorbidities**						
1	1.232 (1.225–1.239)	<0.001	1.207 (1.200–1.213)	<0.001	1.083 (1.079–1.087)	<0.001
2	1.440 (1.431–1.448)	<0.001	1.392 (1.383–1.401)	<0.001	1.145 (1.140–1.149)	<0.001
≥3	1.704 (1.694–1.715)	<0.001	1.635 (1.624–1.646)	<0.001	1.220 (1.214–1.225)	<0.001
**PTB-HTN cluster**						
HH	1.021 (1.015–1.027)	<0.001	1.018 (1.012–1.025)	<0.001	0.946 (0.942–0.950)	<0.001
LL	0.928 (0.901–0.957)	<0.001	0.926 (0.899–0.955)	<0.001	0.937 (0.919–0.955)	<0.001
LH	1.013 (0.99–1.037)	0.272	1.017 (0.994–1.041)	0.152	0.881 (0.868–0.894)	<0.001
**PTB-DM cluster**						
HH	1.185 (1.177–1.192)	<0.001	1.18 (1.173–1.188)	<0.001	1.154 (1.148–1.161)	<0.001
LL	0.854 (0.846–0.863)	<0.001	0.852 (0.843–0.861)	<0.001	0.906 (0.899–0.913)	<0.001
**PTB-COPD cluster**						
HH	0.941 (0.932–0.950)	<0.001	0.939 (0.930–0.948)	<0.001	1.034 (1.027–1.040)	<0.001
LL	1.093 (1.087–1.099)	<0.001	1.106 (1.100–1.112)	<0.001	1.031 (1.027–1.035)	0.002
LH	1.064 (1.043–1.084)	<0.001	1.063 (1.043–1.084)	<0.001	1.088 (1.074–1.101)	<0.001
HL	0.854 (0.829–0.879)	<0.001	0.845 (0.82–0.87)	<0.001	0.833 (0.817–0.848)	<0.001

CI – confidence interval, COPD – chronic obstructive pulmonary disease, DM – diabetes mellitus, exp(*β*) – exponentiated regression coefficients, GLM – generalised linear model, HH – high-high, HL – high-low, LH – low-high, LL – low-low, PTB – pulmonary tuberculosis

**Table 3:** GLM analysis of indirect economic burden for hospitalised PTB patients

	Model 1		Model 2		Model 3	
	**exp(*β*) (95% CI)**	***P*-value**	**exp(*β*) (95% CI)**	***P*-value**	**exp(*β*) (95% CI)**	***P*-value**
**Number of comorbidities**						
1	0.884 (0.878–0.891)	<0.001	1.147 (1.140–1.153)	<0.001	1.146 (1.140–1.152)	<0.001
2	0.702 (0.696–0.708)	<0.001	1.238 (1.230–1.246)	<0.001	1.217 (1.210–1.224)	<0.001
≥3	0.539 (0.535–0.544)	<0.001	1.358 (1.349–1.367)	<0.001	1.302 (1.293–1.310)	<0.001
**PTB-HTN cluster**						
HH	1.118 (1.108–1.127)	<0.001	1.160 (1.152–1.167)	<0.001	0.876 (0.871–0.882)	<0.001
LL	0.834 (0.800–0.870)	<0.001	0.880 (0.854–0.907)	<0.001	0.929 (0.904–0.955)	<0.001
LH	1.993 (1.93–2.058)	<0.001	1.751 (1.711–1.792)	<0.001	1.237 (1.211–1.264)	<0.001
**PTB-DM cluster**						
HH	0.686 (0.680–0.693)	<0.001	0.726 (0.721–0.731)	<0.001	0.688 (0.682–0.694)	<0.001
LL	0.693 (0.684–0.703)	<0.001	0.775 (0.767–0.783)	<0.001	1.361 (1.346–1.375)	<0.001
**PTB-COPD cluster**						
HH	0.638 (0.630–0.647)	<0.001	0.641 (0.635–0.647)	<0.001	0.766 (0.759–0.773)	<0.001
LL	1.766 (1.752–1.78)	<0.001	1.411 (1.403–1.419)	<0.001	1.034 (1.028–1.041)	<0.001
LH	0.806 (0.785–0.828)	<0.001	0.816 (0.801–0.832)	<0.001	0.718 (0.705–0.731)	<0.001
HL	0.528 (0.507–0.549)	<0.001	0.782 (0.759–0.805)	<0.001	0.607 (0.591–0.624)	<0.001

CI – confidence interval, COPD – chronic obstructive pulmonary disease, DM – diabetes mellitus, exp(*β*) – exponentiated regression coefficients, GLM – generalised linear model, HH – high-high, HL – high-low, LH – low-high, LL – low-low, PTB – pulmonary tuberculosis

Economic burden varied across different spatial clustering patterns of comorbidity combinations. For PTB-HTN, compared with areas without significant spatial clustering, patients residing in HH clusters had higher direct economic burden in the unadjusted model (exp(*β*) = 1.021) but lower direct economic burden in the fully adjusted model (exp(*β*) = 0.946). Direct economic burden was consistently lower among patients in LL clusters (exp(*β*) = 0.928–0.937). For LH clusters, no significant difference was observed in the unadjusted model (exp(*β*) = 1.013, *P* = 0.272), whereas lower direct economic burden was observed in the fully adjusted model (exp(*β*) = 0.881, *P* < 0.001). In the sensitivity analysis excluding LOS, direct economic burden remained lower in HH (exp(*β*) = 0.949), LL (exp(*β*) = 0.926), and LH (exp(*β*) = 0.884) clusters than in non-clustered areas (*P* < 0.001), consistent with the direction observed in Model 3. For indirect economic burden, patients in HH clusters had higher burden in the unadjusted model (exp(*β*) = 1.118) but lower burden in the fully adjusted model (exp(*β*) = 0.876). Indirect economic burden was consistently lower in LL clusters (exp(*β*) = 0.834–0.929), whereas it was consistently higher in LH clusters (exp(*β*) = 1.237–1.993).

For PTB-DM, direct economic burden was consistently higher in HH clusters (exp(*β*) = 1.154–1.185, *P* < 0.001) and lower in LL clusters (exp(*β*) = 0.854–0.906; *P* < 0.001) than in non-clustered areas. In the sensitivity analysis excluding LOS, direct economic burden remained higher in HH clusters (exp(*β*) = 1.034, *P* < 0.001), whereas no significant difference was observed for LL clusters (exp(*β*) = 0.992, *P* = 0.190). For indirect economic burden, patients in HH clusters consistently exhibited lower burden (exp(*β*) = 0.686–0.688, *P* < 0.001). In contrast, patients in LL clusters had lower burden in the unadjusted model (exp(*β*) = 0.693, *P* < 0.001) but higher burden in the fully adjusted model (exp(*β*) = 1.361, *P* < 0.001).

For PTB-COPD, direct economic burden in HH clusters was lower than that in non-clustered areas in the unadjusted model (exp(*β*) = 0.941) but became higher after full adjustment (exp(*β*) = 1.034). Direct economic burden was consistently higher in LL clusters (exp(*β*) = 1.031–1.093) and LH clusters (exp(*β*) = 1.064–1.088), whereas it was consistently lower in HL clusters (exp(*β*) = 0.833–0.854). In the sensitivity analysis excluding LOS, direct economic burden remained lower in HH (exp(*β*) = 0.926, *P* < 0.001) and LH (exp(*β*) = 0.954, *P* < 0.001) clusters, remained higher in LL clusters (exp(*β*) = 1.093), and remained lower in HL clusters (exp(*β*) = 0.832). For indirect economic burden, patients in HH clusters consistently exhibited lower burden (exp(*β*) = 0.638–0.766), whereas those in LL clusters consistently exhibited higher burden (exp(*β*) = 1.034–1.766). Lower indirect economic burden was also observed in LH clusters (exp(*β*) = 0.718–0.806) and HL clusters (exp(*β*) = 0.528–0.607) across all models.

Sensitivity analyses were conducted using a Rook contiguity matrix, which defines adjacency based solely on shared boundaries, as a substitute for the Queen contiguity matrix. The direction and statistical significance of the global Moran’s I remained unchanged, and the spatial distributions of different clustering patterns, including HH and LL clusters, were consistent with those obtained using the Queen contiguity matrix and with the main analysis results, indicating good robustness of the findings.

Sensitivity analysis for the economic burden data (reconstructing GLMs using raw cost values) demonstrated findings fully consistent with those reported in the main text (Tables S6 and S7 in the **Online Supplementary Document**). In addition, GLMs based on the complete hospitalisation records data set showed that the directions and statistical significance of the associations for key independent variables remained consistent with the main analysis results, further supporting the robustness of the core conclusions (Tables S8 and S9 in the **Online Supplementary Document**).

In addition, given that LOS may lie on the potential pathway linking comorbidity burden to direct economic burden, a sensitivity analysis was conducted using a Gamma GLM without adjustment for LOS. The direction and statistical significance of the associations between comorbidity count and direct economic burden remained unchanged. The overall patterns of association between spatial clustering of comorbidity combinations and direct economic burden were also largely consistent with those observed in the primary analysis, although the magnitude of the associations varied for some cluster types (Table S5 in the **Online Supplementary Document**). Overall, these findings suggest that the main results were robust to alternative model specifications regarding LOS.

To assess the sensitivity of the findings to within-cluster correlation at the city-year level, the direct and indirect economic burden models were re-estimated using city-year cluster-robust standard errors. The direction, statistical significance, and magnitude of the associations between comorbidity count and economic burden were highly consistent with those observed in the primary analyses. For the spatial clustering variables, the direction and statistical significance of most associations remained stable. Although statistical significance was attenuated for a small number of cluster types, the direction of the associations did not change. Taken together, the principal findings were not materially altered after accounting for city-year clustering, indicating that the results were robust to potential intra-cluster correlation at the city-year level (Table S10 in the **Online Supplementary Document**).

## DISCUSSION

Based on data from 646,114 hospitalised PTB patients in Guangdong Province from 2016 to 2024, we systematically describe the epidemiological characteristics, spatiotemporal distribution patterns, and impact on direct and indirect economic burdens of PTB comorbidities within the inpatient population.

Comorbidity is highly prevalent among hospitalised PTB patients. We found that 66.42% of hospitalised PTB patients had at least one comorbidity, with HTN (18.10%) and DM (16.89%) being the most common. The comorbidity spectrum displayed distinct age stratification. Patients aged <45 years primarily had metabolic and mild respiratory diseases like hyperuricaemia (10.87%) and bronchiectasis (6.00%), those aged 45–60 years saw a marked increase in DM prevalence (23.47%), with chronic diseases like HTN and COPD beginning to emerge, and the ≥60 age group exhibited a typical state of multiple chronic conditions, with significantly higher prevalence of HTN (30.26%), COPD (21.51%), and DM (19.94%). This illustrates an evolution of the comorbidity spectrum from ‘metabolic-mild’ type in younger ages to ‘multiple chronic disease’ type in older ages. Between 2016 and 2024, the comorbidity rate among hospitalised PTB patients increased continuously from 58.55% to 74.86%, with the rate among the ≥60 years group reaching 88.39% in 2024. This reflects a growing burden of comorbidities year by year among hospitalised PTB patients, especially the elderly, and increasing complexity in clinical management. This suggests that future TB control systems urgently need to move beyond single-disease management models towards integrated ‘tuberculosis-comorbidity’ care [11,12]. Systematic screening, assessment, and co-management modules for major chronic diseases (*e.g.* HTN, DM) should be embedded within standardised TB diagnosis and treatment pathways to address the increasingly complex health needs of patients, particularly the elderly [4].

The burden of comorbidities is unevenly distributed within Guangdong Province, with different comorbidity combinations showing significant geographic heterogeneity among hospitalised PTB patients. Spatial analysis showed that statistically significant HH clusters for PTB-HTN were mainly located in the central Pearl River Delta; statistically significant HH clusters for PTB-DM were persistently distributed in Eastern Guangdong; HH clusters for PTB-COPD were mainly located in parts of Eastern and Northern Guangdong. Meanwhile, LL clusters of PTB-DM were consistently observed in western Guangdong, whereas LL clusters of PTB-COPD were relatively stably distributed in several cities of the Pearl River Delta region, suggesting that different comorbidity combinations may have distinct regional epidemiological characteristics and underlying influencing factors. Global Moran's I analysis confirmed statistically significant positive spatial autocorrelation for PTB-HTN and PTB-DM comorbidity rates across all study years, while PTB-COPD comorbidity rates also demonstrated significant spatial clustering in most years, indicating that all three major comorbidity combinations generally exhibited non-random spatial distribution patterns. These spatial clustering phenomena may be associated with regional social determinants of health, including population ageing, baseline chronic disease burden, economic development level, distribution of healthcare resources, and accessibility of health services.

In addition, local spatial association patterns differed across comorbidity combinations. PTB-HTN was mainly characterised by HH and LH clustering patterns, PTB-DM predominantly exhibited HH and LL clustering patterns, whereas PTB-COPD showed multiple local spatial association types, including HH, LL, HL, and LH clusters, indicating a relatively more complex spatial distribution pattern. It should be noted that the hotspot regions we identified were based on statistically significant clustering results from LISA analyses and therefore did not necessarily correspond to areas with simply high comorbidity prevalence. Accordingly, caution is warranted when interpreting local clustering changes, particularly for short-term or single-year clustering variations, which should not be overinterpreted.

Using a GLM analysis, we found that the number of comorbidities was significantly positively correlated with both direct and indirect economic burdens among hospitalised PTB patients, with economic burden progressively increasing as the number of comorbidities increased. This finding is consistent with previous studies examining the economic impacts of multimorbidity in TB patients [13,14]. Multimorbidity may increase hospitalisation expenditures and contribute to productivity losses among hospitalised patients [15]. In our study, the association between the number of comorbidities and economic burden remained stable even after adjustment for demographic characteristics, clinical factors, and regional factors, suggesting that multimorbidity may be an important factor associated with increased economic burden among hospitalised PTB patients.

In the sensitivity analysis of direct economic burden, the association between the number of comorbidities and total hospitalisation costs became stronger after excluding actual hospitalisation days from the model, suggesting that length of hospital stay may partially mediate the relationship between comorbidities and direct economic burden. However, because we used observational data, the specific causal pathways linking comorbidities, disease severity, and healthcare utilisation could not be clearly distinguished, and the underlying mechanisms require further investigation. Regarding indirect economic burden, previous studies have indicated that productivity losses among PTB patients are mainly associated with prolonged hospitalisation, extended treatment duration, and reduced work capacity, while multimorbidity may further exacerbate these economic impacts [16–18]. In our study, an increase in the number of comorbidities was consistently positively associated with higher indirect economic burden, suggesting that multimorbidity may contribute to increased hospitalisation-related productivity losses. Since we estimated indirect economic burden using the human capital approach based on hospitalisation days, the indicator primarily reflects productivity losses during hospitalisation rather than the long-term socioeconomic losses experienced by patients. This limitation is also consistent with previous discussions regarding the challenges of estimating the indirect economic burden of TB [16–18].

The associations between spatial clustering patterns of comorbidity prevalence and economic burden exhibited significant heterogeneity across different comorbidity combinations. For PTB-HTN, HH, LL, and LH clustering areas were all associated with lower direct economic burden in both the fully adjusted models and sensitivity analyses. Among these, the LH clustering areas showed the greatest reduction in direct economic burden after adjustment. Previous studies have reported that HTN prevalence is high among hospitalised PTB patients, significantly exceeding that in the general population [19,20]. Some studies have suggested that HTN is often not systematically screened or managed as an independent comorbidity during clinical care. Due to its insidious symptoms, low patient awareness, and limited primary care resources, HTN management is frequently integrated into routine inpatient care without targeted intervention [21]. Therefore, the lower direct economic burden we observed may be associated with regional differences in comorbidity management patterns, disease severity, or healthcare utilisation structures. Regarding indirect economic burden, LH clustering areas of PTB-HTN remained associated with higher productivity losses after adjustment, whereas HH and LL clustering areas showed negative associations. These findings suggest that hospitalisation-related productivity losses may vary across different spatial clustering patterns. However, because we estimated indirect economic burden based on hospitalisation days, it may not fully capture the actual socioeconomic losses experienced by patients; therefore, these results should be interpreted with caution.

For PTB-DM, HH clustering areas consistently exhibited higher direct economic burden in both the fully adjusted models and sensitivity analyses, whereas LL clustering areas generally showed lower direct economic burden. These findings are broadly consistent with previous studies. Existing evidence suggests that DM may increase disease complexity, the risk of unfavourable treatment outcomes, and healthcare resource utilisation among PTB patients, thereby increasing medical expenditures [22,23]. In our study, HH clustering areas consistently showed lower indirect economic burden in the adjusted models, whereas LL clustering areas shifted to a higher level after adjustment. These findings suggest that hospitalisation-related productivity losses among PTB-DM patients may vary across regions. However, such differences may also be influenced by multiple factors, including age structure, length of hospital stay, disease severity, and regional healthcare service patterns. Therefore, the underlying mechanisms cannot be directly inferred and should be interpreted cautiously.

For PTB-COPD, HH clustering areas were associated with higher direct economic burden in the fully adjusted models; however, this association turned negative in the sensitivity analysis after excluding length of hospital stay, suggesting that length of hospital stay plays an important mediating role between PTB-COPD spatial clustering and direct economic burden. Previous studies have indicated a bidirectional association between PTB and COPD, with comorbid states potentially leading to poorer clinical outcomes and greater healthcare resource utilisation [24–26]. In addition, COPD-related studies have shown that increased disease severity is generally associated with higher direct medical costs and greater productivity losses [27–29]. Therefore, our findings suggest that the higher direct economic burden observed in PTB-COPD high-clustering areas may be related to more complex disease conditions and increased consumption of hospitalisation resources. Regarding indirect economic burden, PTB-COPD HH clustering areas consistently exhibited lower levels, whereas LL clustering areas showed relatively higher levels. These findings were not entirely consistent with the direction of direct economic burden, suggesting that different economic burden indicators may reflect different dimensions of disease impact. On the one hand, the proportion of elderly patients is higher among those with PTB-COPD [24]. Since we estimated hospitalisation-related productivity losses using an age-weighted human capital approach, older patients were assigned relatively lower productivity weights, which may have reduced the overall estimated indirect economic burden. On the other hand, COPD-related studies have shown that productivity losses are generally more pronounced among the working-age population [27,28]. Therefore, the differences in indirect economic burden across clustering areas we observed may be jointly influenced by age structure, labour force participation, and variations in hospitalisation duration, rather than fully reflecting the actual level of socioeconomic losses.

Empirical research shows that current diagnosis-related group/diagnosis-intervention packet payment reforms have achieved significant success in controlling medical costs and improving treatment efficiency for PTB patients [30,31]. Building on this, to incentivise healthcare facilities to provide adequate and appropriate care for TB patients with complex comorbidities (*e.g.* DM, COPD) and avoid under-service due to cost concerns, it is necessary to scientifically evaluate and reasonably adjust the payment weights or scores for such cases within diagnosis-related group/diagnosis-intervention packet reform. Payment standards should better reflect actual resource consumption, guiding the healthcare service system towards integrated and efficient development [31–34]. Given the limited number of DR-TB cases and the primary focus on PTB comorbidity burden, no further subgroup analyses were conducted.

This study has several limitations. First, the analyses were based on hospital discharge records and therefore mainly reflect the comorbidity burden among hospitalised PTB patients, limiting generalisability to all PTB patients. In addition, comorbidity identification relied on inpatient diagnostic records and may be subject to underdiagnosis or coding bias. Second, some conditions, such as hypalbuminaemia and malnutrition, may represent complications or sequelae of PTB rather than independent comorbidities, which could have led to overestimation of the comorbidity burden. However, including these conditions may still help better reflect the long-term health impairment among PTB patients. Third, we estimated indirect economic burden using the human capital approach based on hospitalisation days and mainly reflected hospitalisation-related productivity loss. We did not include other factors, including unemployment, caregiving burden, outpatient costs, long-term disability, and household economic impacts. Finally, the spatial analyses were exploratory in nature, and repeated testing across multiple years and disease combinations may have increased the risk of false-positive local clustering results. In addition, we derived the spatial clustering variables from area-level LISA analyses and primarily reflect regional patterns of comorbidity prevalence and spatial concentration. Therefore, the observed associations between spatial clustering patterns and economic burden should be interpreted as relationships between regional contextual characteristics and individual economic burden rather than evidence of causality. As with all observational studies, residual confounding may remain. Future studies incorporating prospective designs, multilevel modelling approaches, and indicators of regional healthcare resources are needed to further elucidate the underlying mechanisms.

## CONCLUSIONS

Based on hospitalisation big data from Guangdong Province during 2016–2024, we found that comorbidity burden was positively associated with economic burden among hospitalised patients with PTB. Associations between spatial clustering patterns of PTB comorbidity combinations and economic burden were heterogeneous, with economic burden varying across different cluster types. These findings highlight the importance of considering both individual comorbidity burden and regional comorbidity patterns when developing PTB comorbidity management and resource allocation strategies. Integrated management of PTB and chronic diseases, together with optimised healthcare resource allocation and interventions addressing both direct medical expenditures and productivity losses, may contribute to reducing the socioeconomic burden of PTB. These results provide important evidence for optimising inpatient services and implementing regionalised in-hospital comorbidity management and resource allocation. Future policies for PTB comorbidity prevention and control should transcend the traditional ‘cost-effectiveness’ framework and move towards a comprehensive management strategy aimed at ‘minimising total social costs’.

## Additional material


Online Supplementary Document

## Data Availability

**Data availability:** For data security reasons, all data were analysed in an intranet database, and raw data are not publicly available. De-identified data are available from the corresponding author upon reasonable request.
