# Supplementary material for: Spatial distribution, clustering patterns, and economic burden of comorbidities among hospitalised pulmonary tuberculosis patients in Guangdong province: a spatial epidemiological study based on inpatient big data
Source: J Glob Health. 2026 Aug 28;16:04284. doi: 10.7189/jogh.16.04284 (PMC13523040; doi:10.7189/jogh.16.04284)
Supplement: Supplementary file 1 [file jogh-16-04284-s001.pdf]

Supplement to: Ji L, Chen L, Xue Y, Wu Y, Wu HZ, Jiang X, Liu G, Wang X, Yuan S, Zhou L. Spatial Distribution, clustering patterns, and economic burden of comorbidities among hospitalised pulmonary tuberculosis patients in Guangdong Province: a spatial epidemiological study based on inpatient big data. J Glob Health. 2026;16:04284.

Table S1: Comorbidities included in the analysis and their ICD-10 codes

Disease Name	ICD-10 Code(s)	Disease Name	ICD-10 Code(s)
Hypertension	I10-I15	Atherosclerosis	I70
Diabetes mellitus	E10-E14	Mental and behavioral disorders	F00-F99
Malignant neoplasms	C00-C97	Hyperuricemia	E79.001
Rheumatoid arthritis	M05-M06	Bronchiectasis	J47
Hypoproteinemia	E77.801	Chronic kidney disease	N18
Asthma	J45-J46	Emphysema	J43
Viral hepatitis	B15-B19	Malnutrition	E40-E46
Cerebrovascular diseases	I60-I69	Coronary atherosclerosis	I20-I25
Chronic obstructive pulmonary disease	J44	Human immunodeficiency virus [HIV] disease	B20-B24
Liver cirrhosis	A52.705+K77.0, B65.202+K77.0, K70.200, K70.300-K70.302+I98.3, K71.700-K71.702, K73.900x001, K74.100-K74.622, K76.101, O26.604, P78.803	Disorders of lipoprotein metabolism and other lipidemias	E78

Table S2: Distribution of comorbidity types among hospitalized PTB patients by age group (n, %)

**Comorbidity Disease**	**Total Comorbid Cases (Comorbidity Rate)**	**<45 years**	**45–60 years**	**≥60 years**
Hypertension	116915(18.10)	3794(2.07)	23947(14.28)	89174(30.26)
Diabetes mellitus	109133(16.89)	10987(5.98)	39373(23.47)	58773(19.94)
COPD	73633(11.40)	725(0.39)	9498(5.66)	63410(21.51)
Hypoproteinemia	72127(11.16)	11649(6.34)	16215(9.67)	44263(15.02)
Bronchiectasis	68116(10.54)	11015(6.00)	16732(9.97)	40369(13.70)
Cerebrovascular diseases	63592(9.84)	1364(0.74)	9741(5.81)	52487(17.81)
Emphysema	60749(9.40)	5368(2.92)	15317(9.13)	40064(13.59)
Hyperuricemia	56297(8.71)	19958(10.87)	14631(8.72)	21708(7.37)
Coronary atherosclerotic heart disease	41946(6.49)	564(0.31)	6143(3.66)	35239(11.96)
Atherosclerosis	37313(5.77)	557(0.30)	7197(4.29)	29559(10.03)
Malignant tumor	35055(5.43)	2258(1.23)	8995(5.36)	23802(8.08)
Disorders of lipoprotein metabolism and other lipidemias	34390(5.32)	5319(2.90)	12042(7.18)	17029(5.78)
Viral hepatitis	27876(4.31)	9303(5.07)	11026(6.57)	7547(2.56)
Chronic kidney disease	17994(2.78)	1437(0.78)	4218(2.51)	12339(4.19)
Mental and behavioral disorders	12628(1.95)	3555(1.94)	3555(2.12)	5518(1.87)
Malnutrition	12480(1.93)	2682(1.46)	2479(1.48)	7319(2.48)
Liver cirrhosis	11137(1.72)	1238(0.67)	4745(2.83)	5154(1.75)
Human Immunodeficiency Virus [HIV] Disease	116915(0.84)	2320(1.26)	1840(1.10)	1263(0.43)
Rheumatoid arthritis	5043(0.78)	385(0.21)	1594(0.95)	3064(1.04)
Asthma	4122(0.64)	650(0.35)	1338(0.80)	2134(0.72)

Table S3: Comparison of comorbidity rates among hospitalized PTB patients with different demographic characteristics

Characteristic	Group	N (Total)	N with Comorbidity	Comorbidity Rate (%)	χ^2 value	P value
Sex						
	Male	476826	334067	70.06	10790.154	<0.001
	Female	169288	95109	56.18		
Age						
	<45 years	183637	68546	37.33	105598.17	<0.001
	45~60 years	167749	114292	68.13		
	≥60 years	294728	246338	83.58		

Table S4: Spatial autocorrelation analysis of the top three comorbidity combinations, 2016-2024

year	PTB-HTN			PTB-DM			PTB-COPD		
	**Moran's I**	**Z**	***P***	**Moran's I**	**Z**	***P***	**Moran's I**	**Z**	***P***
2016	0.232	1.962	0.033	0.425	3.066	0.002	0.384	2.671	0.008
2017	0.293	2.366	0.014	0.301	2.277	0.017	0.381	2.645	0.006
2018	0.283	2.306	0.016	0.344	2.590	0.005	0.456	3.114	0.002
2019	0.274	2.250	0.019	0.460	3.307	0.003	0.379	2.702	0.004
2020	0.227	1.909	0.044	0.494	3.458	0.001	0.297	2.260	0.017
2021	0.276	2.148	0.027	0.378	2.762	0.004	0.278	2.136	0.021
2022	0.242	1.959	0.037	0.500	3.434	0.001	0.296	2.205	0.021
2023	0.244	2.007	0.033	0.460	3.238	0.002	0.332	2.433	0.010
2024	0.401	2.985	0.002	0.452	3.159	0.003	0.215	1.672	0.061

Table S5: Sensitivity Analysis: Generalized Linear Model(GLM) of direct economic burden in hospitalized pulmonary tuberculosis patients (excluding actual hospitalization days)

Parameter	Model 3	
	**Exp (B) (95 %CI)**	**P**
Number of comorbidities		
Only 1 comorbidity	1.192(1.187,1.198)	<0.001
Only 2 comorbidities	1.308(1.301,1.316)	<0.001
≥3 comorbidities	1.449(1.44,1.457)	<0.001
PTB-HTN		
High-high cluster	0.949(0.944,0.954)	<0.001
Low-low cluster	0.926(0.902,0.95)	<0.001
Low-high cluster	0.884(0.867,0.902)	<0.001
PTB-DM		
High-high cluster	1.034(1.026,1.042)	<0.001
Low-low cluster	0.992(0.982,1.002)	0.19
PTB-COPD		
High-high cluster	0.926(0.919,0.934)	<0.001
Low-low cluster	1.093(1.086,1.099)	<0.001
Low-high cluster	0.954(0.939,0.97)	<0.001
High-low cluster	0.832(0.811,0.853)	<0.001

Table S6: Sensitivity analysis: GLM assessing the direct economic burden of hospitalized pulmonary tuberculosis patients using raw data

Parameter	Model 1		Model 2		Model 3	
	Exp (B) (95 %CI)	P	Exp (B) (95 %CI)	P	Exp (B) (95 %CI)	P
Number of comorbidities						
Only 1 comorbidity	1.232(1.225,1.239)	<0.001	1.207(1.2,1.213)	<0.001	1.083(1.079,1.087)	<0.001
Only 2 comorbidities	1.44(1.431,1.448)	<0.001	1.392(1.383,1.401)	<0.001	1.145(1.14,1.149)	<0.001
≥3 comorbidities	1.704(1.694,1.715)	<0.001	1.635(1.624,1.646)	<0.001	1.22(1.214,1.225)	<0.001
PTB-HTN						
High-high cluster	1.021(1.015,1.027)	<0.001	1.018(1.012,1.025)	<0.001	0.946(0.942,0.95)	<0.001
Low-low cluster	0.928(0.901,0.957)	<0.001	0.926(0.899,0.955)	<0.001	0.937(0.919,0.955)	<0.001
Low-high cluster	1.013(0.99,1.037)	0.272	1.017(0.994,1.041)	0.152	0.881(0.868,0.894)	<0.001
PTB-DM						
High-high cluster	1.185(1.177,1.192)	<0.001	1.18(1.173,1.188)	<0.001	1.154(1.148,1.161)	<0.001
Low-low cluster	0.854(0.846,0.863)	<0.001	0.852(0.843,0.861)	<0.001	0.906(0.899,0.913)	<0.001
PTB-COPD						
High-high cluster	0.941(0.932,0.95)	<0.001	0.939(0.93,0.948)	<0.001	1.034(1.027,1.04)	<0.001
Low-low cluster	1.093(1.087,1.099)	<0.001	1.106(1.1,1.112)	<0.001	1.031(1.027,1.035)	0.002
Low-high cluster	1.064(1.043,1.084)	<0.001	1.063(1.043,1.084)	<0.001	1.088(1.074,1.101)	<0.001
High-low cluster	0.854(0.829,0.879)	<0.001	0.845(0.82,0.87)	<0.001	0.833(0.817,0.848)	<0.001

Table S7: Sensitivity analysis: GLM assessing the indirect economic burden of hospitalized pulmonary tuberculosis patients using raw data

Parameter	Model 1		Model 2		Model 3	
	Exp (B) (95 %CI)	P	Exp (B) (95 %CI)	P	Exp (B) (95 %CI)	P
Number of comorbidities						
Only 1 comorbidity	0.884(0.878,0.891)	<0.001	1.147(1.14,1.153)	<0.001	1.146(1.14,1.152)	<0.001
Only 2 comorbidities	0.702(0.696,0.708)	<0.001	1.238(1.23,1.246)	<0.001	1.217(1.21,1.224)	<0.001
≥3 comorbidities	0.539(0.535,0.544)	<0.001	1.358(1.349,1.367)	<0.001	1.302(1.293,1.31)	<0.001
PTB-HTN						
High-high cluster	1.118(1.108,1.127)	<0.001	1.16(1.152,1.167)	0.096	0.876(0.871,0.882)	<0.001
Low-low cluster	0.834(0.8,0.87)	<0.001	0.88(0.854,0.907)	<0.001	0.929(0.904, 0.955)	<0.001
Low-high cluster	1.993(1.93,2.058)	<0.001	1.751(1.711,1.792)	<0.001	1.237(1.211,1.264)	<0.001
PTB-DM						
High-high cluster	0.686(0.68,0.693)	<0.001	0.726(0.721,0.731)	<0.001	0.688(0.682,0.694)	<0.001
Low-low cluster	0.693(0.684,0.703)	<0.001	0.775(0.767,0.783)	<0.001	1.361(1.346,1.375)	<0.001
PTB-COPD						
High-high cluster	0.638(0.63,0.647)	<0.001	0.641(0.635,0.647)	<0.001	0.766(0.759,0.773)	<0.001
Low-low cluster	1.766(1.752,1.78)	<0.001	1.411(1.403,1.419)	<0.001	1.034(1.028,1.041)	<0.001
Low-high cluster	0.806(0.785,0.828)	<0.001	0.816(0.801,0.832)	<0.001	0.718(0.705,0.731)	<0.001
High-low cluster	0.528(0.507,0.549)	<0.001	0.782(0.759,0.805)	<0.001	0.607(0.591,0.624)	<0.001

Table S8: Sensitivity Analysis: Generalized Linear Model (GLM) for assessing the direct economic burden of hospitalized pulmonary tuberculosis patients using all hospitalization records

Parameter	Model 1		Model 2		Model 3	
	**Exp (B) (95 %CI)**	**P**	**Exp (B) (95 %CI)**	**P**	**Exp (B) (95 %CI)**	**P**
Number of comorbidities						
Only 1 comorbidity	1.246(1.241,1.252)	<0.001	1.23(1.224,1.235)	<0.001	1.085(1.082,1.089)	<0.001
Only 2 comorbidities	1.458(1.451,1.466)	<0.001	1.428(1.42,1.436)	<0.001	1.146(1.142,1.15)	<0.001
≥3 comorbidities	1.713(1.704,1.722)	<0.001	1.669(1.66,1.679)	<0.001	1.217(1.213,1.222)	<0.001
PTB-HTN						
High-high cluster	1.023(1.018,1.028)	<0.001	1.021(1.016,1.027)	<0.001	0.945(0.942,0.949)	<0.001
Low-low cluster	0.923(0.901,0.946)	<0.001	0.922(0.899,0.945)	<0.001	0.968(0.953,0.983)	<0.001
Low-high cluster	1.025(1.005,1.046)	0.013	1.027(1.007,1.048)	0.008	0.875(0.864,0.886)	<0.001
PTB-DM						
High-high cluster	1.184(1.177,1.191)	<0.001	1.181(1.175,1.188)	<0.001	1.156(1.151,1.162)	<0.001
Low-low cluster	0.859(0.841,0.866)	<0.001	0.857(0.85,0.865)	<0.001	0.895(0.889,0.901)	<0.001
PTB-COPD						
High-high cluster	0.953(0.945,0.961)	<0.001	0.952(0.944,0.959)	<0.001	1.038(1.032,1.043)	<0.001
Low-low cluster	1.105(1.099,1.11)	<0.001	1.113(1.107,1.118)	<0.001	1.023(1.019,1.027)	0.002
Low-high cluster	1.073(1.055,1.092)	<0.001	1.073(1.055,1.091)	<0.001	1.077(1.066,1.089)	<0.001
High-low cluster	0.848(0.826,0.869)	<0.001	0.842(0.821,0.863)	<0.001	0.828(0.814,0.841)	<0.001

Table S9: Sensitivity Analysis: Generalized Linear Model (GLM) for assessing the indirect economic burden of hospitalized pulmonary tuberculosis patients using all hospitalization records

Parameter	Model 1		Model 2		Model 3	
	**Exp (B) (95 %CI)**	**P**	**Exp (B) (95 %CI)**	**P**	**Exp (B) (95 %CI)**	**P**
Number of comorbidities						
Only 1 comorbidity	0.897(0.892,0.903)	<0.001	1.162(1.157,1.168)	<0.001	1.148(1.143,1.153)	<0.001
Only 2 comorbidities	0.729(0.724,0.735)	<0.001	1.271(1.264,1.278)	<0.001	1.227(1.221,1.233)	<0.001
≥3 comorbidities	0.572(0.568,0.576)	<0.001	1.415(1.407,1.423)	<0.001	1.325(1.318,1.332)	<0.001
PTB-HTN						
High-high cluster	1.111(1.103,1.119)	<0.001	1.162(1.156,1.168)	0.096	0.856(0.851,0.86)	<0.001
Low-low cluster	0.821(0.794,0.85)	<0.001	0.867(0.845,0.889)	<0.001	0.925(0.904, 0.947)	<0.001
Low-high cluster	1.96(1.907,2.014)	<0.001	1.769(1.734,1.804)	<0.001	1.218(1.196,1.241)	<0.001
PTB-DM						
High-high cluster	0.68(0.675,0.686)	<0.001	0.706(0.702,0.71)	<0.001	0.695(0.69,0.701)	<0.001
Low-low cluster	0.711(0.703,0.72)	<0.001	0.776(0.769,0.783)	<0.001	1.338(1.325,1.35)	<0.001
PTB-COPD						
High-high cluster	0.65(0.642,0.657)	<0.001	0.647(0.642,0.653)	<0.001	0.777(0.771,0.783)	<0.001
Low-low cluster	1.783(1.771,1.795)	<0.001	1.423(1.416,1.43)	<0.001	1.013(1.007,1.018)	<0.001
Low-high cluster	0.822(0.803,0.841)	<0.001	0.833(0.819,0.847)	<0.001	0.737(0.725,0.749)	<0.001
High-low cluster	0.555(0.536,0.575)	<0.001	0.779(0.76,0.799)	<0.001	0.595(0.581,0.609)	<0.001

Table S10: Sensitivity Analysis of Direct and Indirect Economic Burden Models Using City-Year Clustered Robust Standard Errors

Parameter	Direct Economic Burden		indirect Economic Burden	
	**Exp (B) (95 %CI)**	**P**	**Exp (B) (95 %CI)**	**P**
Number of comorbidities				
Only 1 comorbidity	1.077(1.068,1.085)	<0.001	1.152(1.138,1.167)	<0.001
Only 2 comorbidities	1.133(1.121,1.146)	<0.001	1.228(1.205,1.251)	<0.001
≥3 comorbidities	1.2(1.183,1.217)	<0.001	1.321(1.285,1.357)	<0.001
PTB-HTN				
High-high cluster	0.947(0.91,0.984)	0.006	0.875(0.78,0.982)	0.023
Low-low cluster	0.811(0.788,0.835)	<0.001	0.957(0.911, 1.005)	0.077
Low-high cluster	0.874(0.793,0.964)	0.007	1.231(1.077,1.406)	0.002
PTB-DM				
High-high cluster	1.138(1.06,1.222)	<0.001	0.688(0.602,0.786)	<0.001
Low-low cluster	0.931(0.875,0.991)	0.024	1.336(1.224,1.458)	<0.001
PTB-COPD				
High-high cluster	1.044(0.977,1.116)	0.202	0.77(0.671,0.884)	<0.001
Low-low cluster	1.044(1.004,1.085)	0.031	1.038(0.911,1.182)	0.579
Low-high cluster	1.116(1.038,1.201)	0.003	0.69(0.594,0.801)	<0.001
High-low cluster	0.744(0.72,0.769)	<0.001	0.615(0.555,0.682)	<0.001

Figure S1. Numbers and Costs of Hospitalized Pulmonary Tuberculosis Patients, and Numbers of Hospitalized Drug-Resistant Tuberculosis Patients. **Panel A.** Trends in the number of hospitalized patients with pulmonary tuberculosis and those with drug-resistant tuberculosis from 2016 to 2024**; Panel B.** Expenditure composition for hospitalized pulmonary tuberculosis patients, including direct economic burden, out-of-pocket costs, indirect economic burden, out-of-pocket proportion, and medication cost proportion.

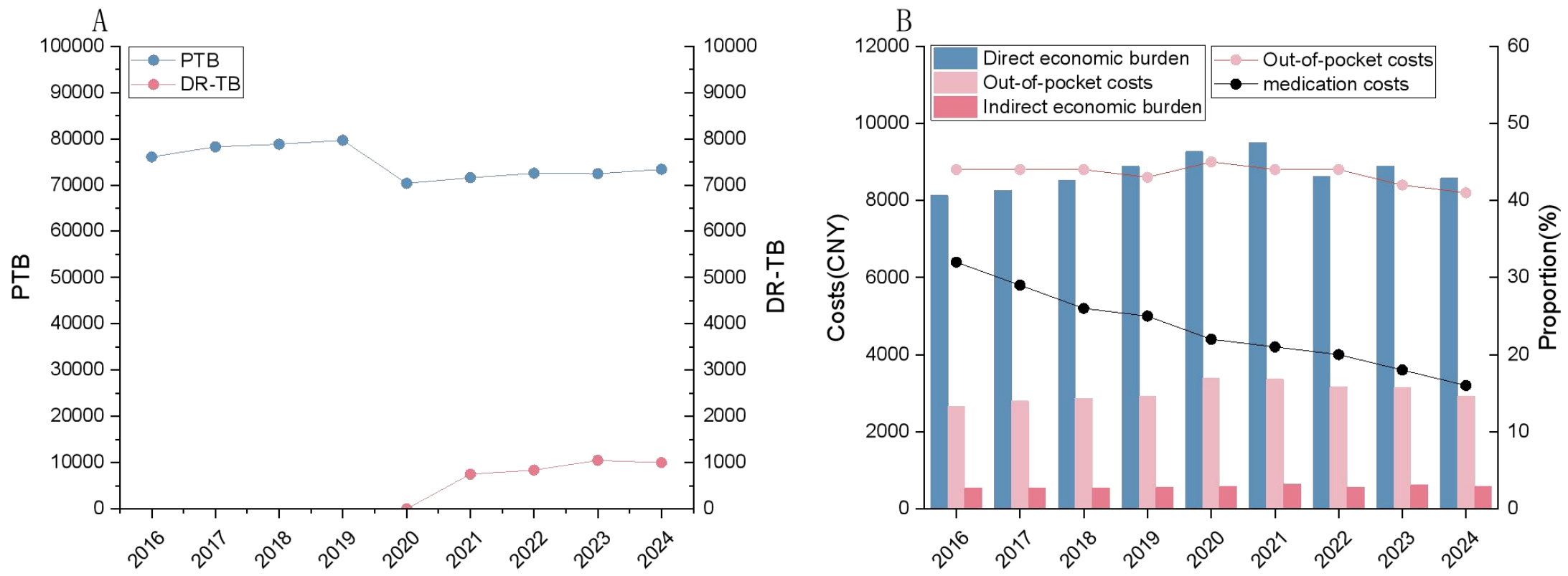

Figure S2. Comorbidity patterns of major comorbidity combinations among hospitalized patients with pulmonary tuberculosis. **Panel A.** Temporal changes in the number and prevalence of major comorbidity combinations from 2016 to 2024. **Panel B.** Heat map of the comorbidity prevalence of HTN among hospitalized PTB patients across cities in Guangdong Province. **Panel C.** Heat map of the comorbidity prevalence of DM among hospitalized PTB patients across cities in Guangdong Province. **Panel D.** Heat map of the comorbidity prevalence of COPD among hospitalized PTB patients across cities in Guangdong Province. HTN Hypertension; DM Diabetes mellitus; COPD Chronic obstructive pulmonary disease

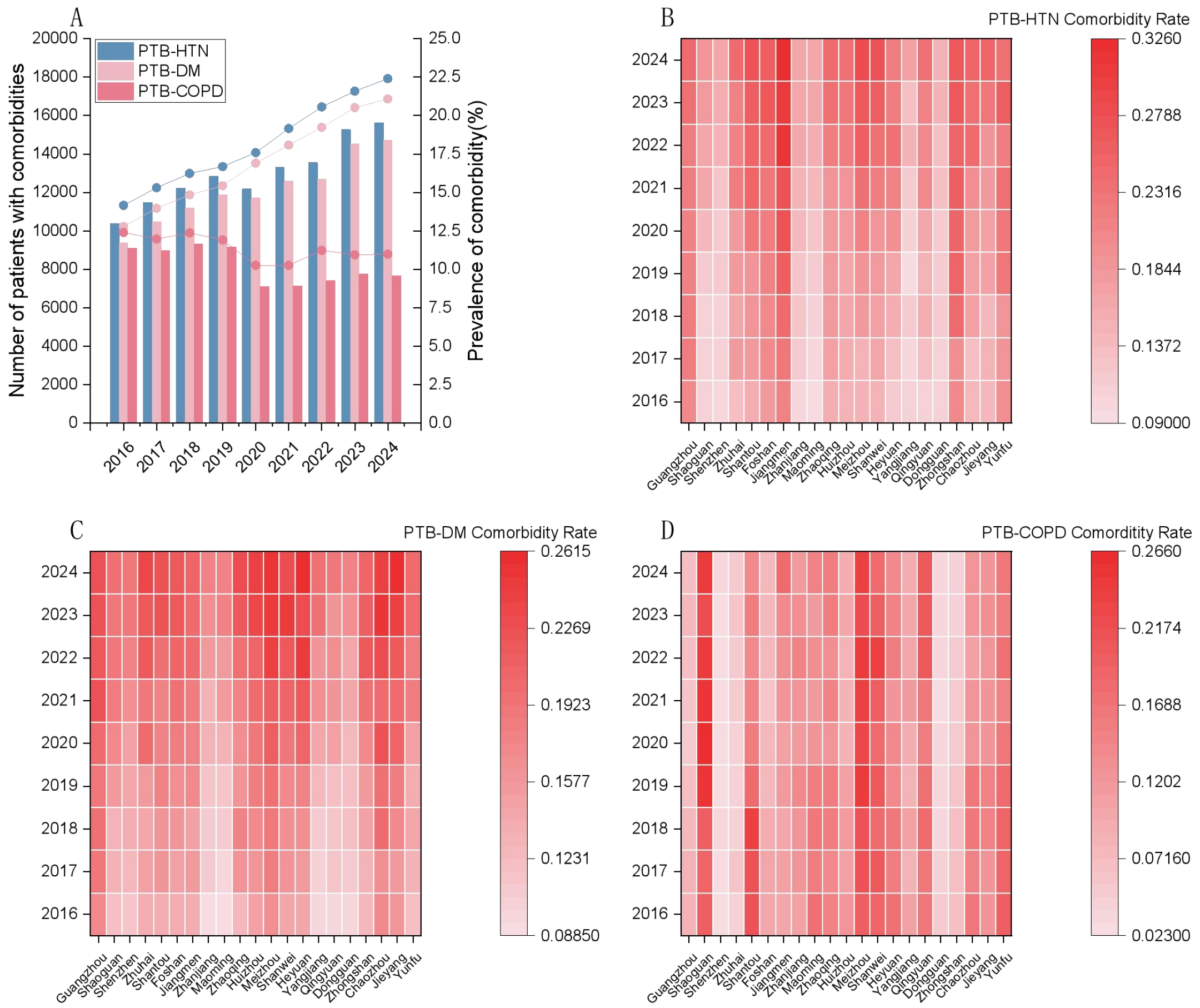

STROBE Statement—Checklist of items that should be included in reports of ***cross-sectional studies***

	Item No	Recommendation	Page No
**Title and abstract**	1	(*a*) Indicate the study's design with a commonly used term in the title or the abstract	1
		(*b*) Provide in the abstract an informative and balanced summary of what was done and what was found	2
**Introduction**			
Background/rationale	2	Explain the scientific background and rationale for the investigation being reported	3-4
Objectives	3	State specific objectives, including any prespecified hypotheses	4-5
**Methods**			
Study design	4	Present key elements of study design early in the paper	5-6
Setting	5	Describe the setting, locations, and relevant dates, including periods of recruitment, exposure, follow-up, and data collection	5-6
Participants	6	(*a*) Give the eligibility criteria, and the sources and methods of selection of participants	5-6
Variables	7	Clearly define all outcomes, exposures, predictors, potential confounders, and effect modifiers. Give diagnostic criteria, if applicable	6-7
Data sources/ measurement	8*	For each variable of interest, give sources of data and details of methods of assessment (measurement). Describe comparability of assessment methods if there is more than one group	5-9
Bias	9	Describe any efforts to address potential sources of bias	9-10
Study size	10	Explain how the study size was arrived at	10
Quantitative variables	11	Explain how quantitative variables were handled in the analyses. If applicable, describe which groupings were chosen and why	6-7
Statistical methods	12	(*a*) Describe all statistical methods, including those used to control for confounding	7-10
		(*b*) Describe any methods used to examine subgroups and interactions	7-10
		(*c*) Explain how missing data were addressed	5
		(*d*) If applicable, describe analytical methods taking account of sampling strategy	7-10
		(*e*) Describe any sensitivity analyses	9-10
**Results**			
Participants	13*	(a) Report numbers of individuals at each stage of study—eg numbers potentially eligible, examined for eligibility, confirmed eligible, included in the study, completing follow-up, and analysed	10
		(b) Give reasons for non-participation at each stage	N/A
		(c) Consider use of a flow diagram	N/A
Descriptive data	14*	(a) Give characteristics of study participants (eg demographic, clinical, social) and information on exposures and potential confounders	10-12
		(b) Indicate number of participants with missing data for each variable of interest	N/A

Outcome data	15*	Report numbers of outcome events or summary measures	10-16
Main results	16	(*a*) Give unadjusted estimates and, if applicable, confounder-adjusted estimates and their precision (eg, 95% confidence interval). Make clear which confounders were adjusted for and why they were included	16-20
		(*b*) Report category boundaries when continuous variables were categorized	6
		(*c*) If relevant, consider translating estimates of relative risk into absolute risk for a meaningful time period	N/A
Other analyses	17	Report other analyses done—eg analyses of subgroups and interactions, and sensitivity analyses	18-20
**Discussion**			
Key results	18	Summarise key results with reference to study objectives	20-25
Limitations	19	Discuss limitations of the study, taking into account sources of potential bias or imprecision. Discuss both direction and magnitude of any potential bias	25-26
Interpretation	20	Give a cautious overall interpretation of results considering objectives, limitations, multiplicity of analyses, results from similar studies, and other relevant evidence	21-26
Generalisability	21	Discuss the generalisability (external validity) of the study results	25-26
**Other information**			
Funding	22	Give the source of funding and the role of the funders for the present study and, if applicable, for the original study on which the present article is based	27

*Give information separately for exposed and unexposed groups.

Note: An Explanation and Elaboration article discusses each checklist item and gives methodological background and published examples of transparent reporting. The STROBE checklist is best used in conjunction with this article (freely available on the Web sites of PLoS Medicine at http://www.plosmedicine.org/, Annals of Internal Medicine at http://www.annals.org/, and Epidemiology at http://www.epidem.com/). Information on the STROBE Initiative is available at www.strobe-statement.org.
